# Characterization of the physicochemical properties, antioxidant activity, and antiproliferative activity of natural melanin from *S. reiliana*

**DOI:** 10.1038/s41598-022-05676-z

**Published:** 2022-02-08

**Authors:** Xin Fu, Mengxi Xie, Ming Lu, Lin Shi, Taiyuan Shi, Miao Yu

**Affiliations:** 1 Food and Processing Research Institute, Liaoning Academy of Agricultural Sciences, Shenyang, China; 2 College of Food Science, Shenyang Agricultural University, Shenyang, China

**Keywords:** Fungi, Industrial microbiology

## Abstract

This study aimed to characterize the physicochemical properties and stability of L-25 melanin extracted from *Sporisorium reilianum* (*S.* reiliana). The results showed that the maximum absorption wavelength of melanin was 215 nm. Reducing agents, heat, light, microwaving, oxidants, and common food additives did not affect the melanin. Additionally, it has a good metal stability except Mn^2+^. The IR spectra revealed the presence of O–H, N–H, C=O, and C=C bonds as well as carboxyl, alcohol hydroxyl, and phenolic hydroxyl groups and a pyran ring. L-25 melanin could be defined as DL-hydroxy phenylalanine (DOPA)-melanin. The antioxidant and antiproliferative were also measured. The melanin has a specific stability and high antioxidant activity, including a strong DPPH free radical scavenging ability, and protected damaged HepG2 cells by reducing reactive oxygen species, malondialdehyde, and lactate dehydrogenase content. In conclusion, *S. reilianum* represents a novel source of melanin, that could be applied to health food or food additives. Our results show that melanin from *S. reilianum* is a natural pigment with good stability that has a great prospect of development and application, providing a theoretical basis and methods for its further processing and development as a functional food.

## Introduction

Melanin is an irregular pigment that is widely present in various organisms^1,2^. The pigment has a wide range of biological effects, including antioxidant, antitumor, antivenin, and radiation resistance activity^3,4^. In recent years, melanin and melanin-like molecules have been widely explored in biomedical applications such as bioimaging, photothermal therapy, and drug delivery systems, and are also widely used in many fields as a functional material due to their excellent biocompatibility and biodegradability^1–8^. Many macrofungi produce melanin, which makes fungi an important source of melanin. Melanin has two synthetic pathways in fungi: some fungi use the 1,8-dihydroxy naphthalene (DHN) synthesis pathway while other fungi use the 3,4-dihydroxyphenylalanine (DOPA) synthesis pathway^7,9^. In the DHN synthesis pathway, the formation of tetrahydroxy naphthalene (1,3,6,8-THN) occurs through the action of polyketide synthetase, followed by a series of reactions leading to the formation of DNH, which is further polymerized to form melanin. In the fungal DOPA pathway, which is similar to the mammalian melanin biosynthesis pathway, 5-hydroxy indoles are synthesized by a series of reactions with L-DOPA or tyrosine as the starting molecule and these 5-hydroxy indoles are then polymerized to form melanin. Melanin can be used not only as natural melanin in the food industry but also in the cosmetics industry to prepare products such as hair dyes and sunscreen. Biological pesticides are being developed in agriculture, while antiviral and antifungal drugs are being developed in the medical field^2,10^*.*

*S. reiliana*, a typical black food and edible fungus, not only has high melanin content but also has many positive effects on human health^11,12^. It is widely used in common traditional Chinese medicines and has many applications, including regulating menstruation, stopping bleeding, reducing blood cholesterol, controlling body weight, and preventing colitis and coronary heart disease^13,14^. The melanin contained in mature *S. reiliana* is usually a macromolecule formed by oxidative polymerization of indole or phenolic compounds^15^, which can protect organisms from environmental stress^16^. In China, it is widely used in traditional Chinese medicines that are commonly used in the private sector. It has many effects, such as regulating menstruation, hemostasis, lowering blood cholesterol, weight control, and preventing colitis and coronary heart disease. However, the research focus on *S. reiliana* is mainly as a plant pathogen infection mechanism and classification. In recent years, active metabolites of *S. reiliana* have been studied extensively, and multiple new active compounds have been identified^2^. Although melanin is an important active ingredient in *S. reiliana*, its active fractions and chemical structures related to antioxidative effects have rarely been addressed.

In this paper, we studied the physicochemical properties of L-25 melanin that was extracted from *S. reiliana* as previously reported^3^. The melanin was analyzed by scanning electron microscopy (SEM), dynamic light scattering (ultra-performance liquid chromatography (UPLC)-quadrupole time of flight mass spectrometry (QTOF-MS)), elemental analysis, ultraviolet–visible spectrometry (UV–Vis), and infrared spectrometry (IR). Furthermore, the antioxidative effects and antiproliferative activities of the purified melanin were studied. Our results provide insights into the physicochemical properties of melanin extracted from *S. reiliana* and its potential use in functional foods or food additives.

## Results and discussion

### Physicochemical properties of L-25 melanin

As shown in supplementary materials (Table S1), L-25 melanin had relatively low solubility in water, acid, and most organic solvents including ethanol, dichloromethane, ethyl acetate, acetonitrile, and acetone. However, in strong alkaline solutions, such as NaOH, the solubility was higher. The melanin was slightly soluble in dimethyl sulfoxide and methanol. It can precipitate out in a strong acidic solution. This solubility characteristic of L-25 melanin was very similar to melanin obtained from other microorganisms.

The effects of the environmental conditions including illumination, temperature, microwaves, metal ions, oxidizers, and reducers on the stability of L-25 melanin were also studied. The pH value strongly influences the melanin of sorghum black powder fungus, including changes in light absorption value and color (Table S2). The melanin appeared brownish red in the range of pH 2–7 and turned dark brown when pH > 8. The effect of light on melanin was not obvious, but with prolonged exposure to natural light or ultraviolet light irradiation, there was a slight increase in the light absorption value (Table S3). The reason may be the enhancement of the polymerization of melanin from sorghum during exposure to light. We also speculated that L-25 melanin has a certain protective effect against ultraviolet radiation. With the extension of microwave time from 0 to 30 min, the absorbance of melanin hardly changed, indicating that microwave treatment had little effect on L-25 melanin (Table S4). Our study also found that treatment with the reducing agent (Na_2_SO_3_, VC and sodium benzoate) had no significant effect on the stability of L-25 melanin. Moreover, the reducing agent Na_2_SO_3_ had no significant effect on the stability of L-25 melanin. However, the oxidizing agent H_2_O_2_ reduced the stability of IH melanin (Tables S5–S7).

**Figure 1:**
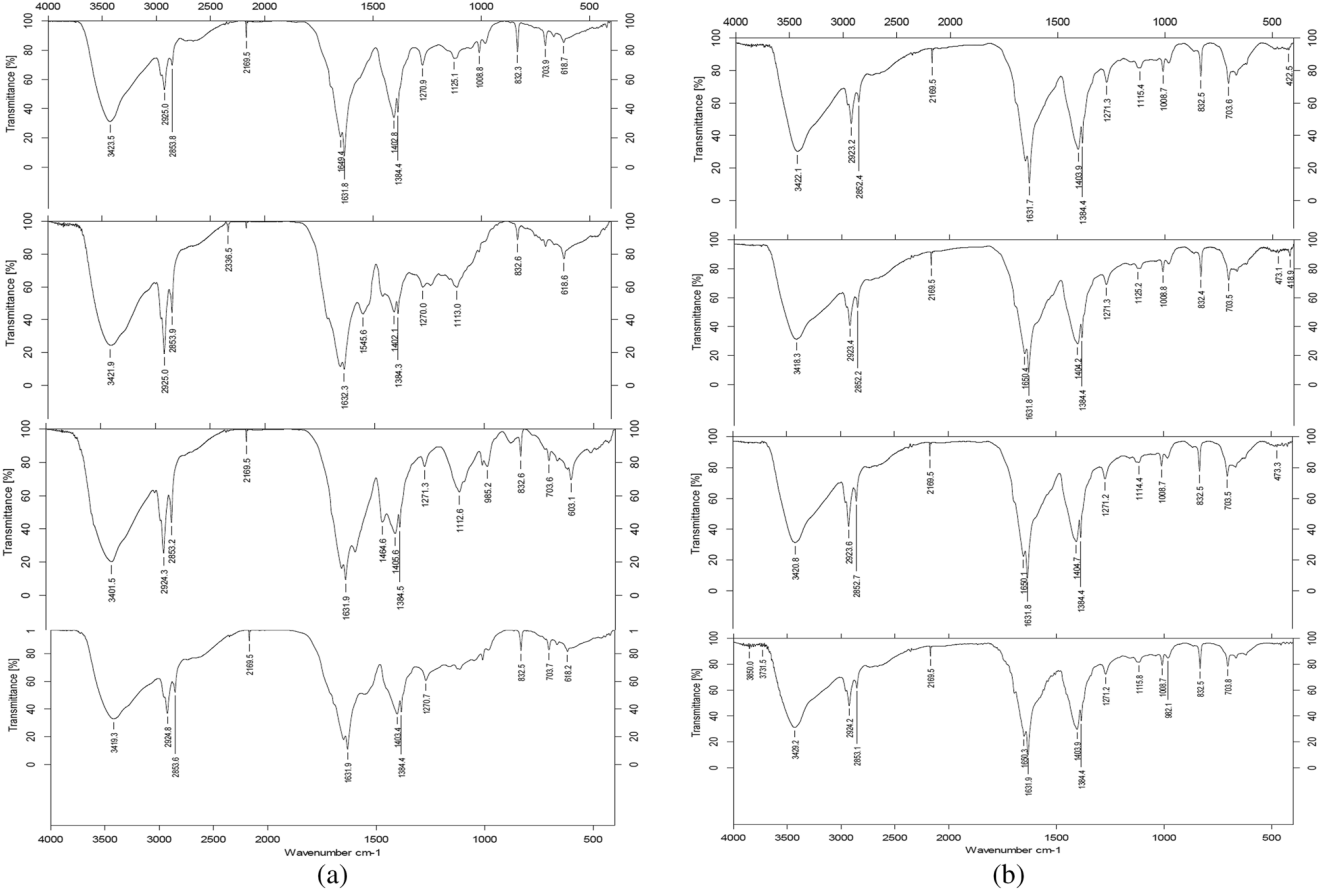
(**a**) IR spectra of the melanin from head smut with Cu^2+^ ions for the different pH values. (**b**) IR spectra of the melanin from head smut with Fe^3+^ ions for the different pH values.

Among the metal ions used, Mn^2+^ had the most significant impact on the stability of L-25 melanin (Table 1), which may be related to complex reactions between the metal ions and several neighboring phenolic hydroxyl groups of melanin^9,17^. The remaining metal ions had no significant effect on the stability of the melanin compared with the control. Cu^2+^ and Fe^3+^ play a very important role in the formation of melanin. The active site of tyrosinase, a key enzyme in the process of eumelanin formation, contains both a Cu^2+^ and Fe^3+^ ion. The infrared spectrum characteristics of complexes between metal ions Cu^2+^, and Fe^3+^ and L-25 melanin were compared to that of sorghum melanin not complexed to a metal ion (Fig. 1). We found that the pH value of the complex and the solution affected the complexation of melanin and metal ions. The effect on the complex sum of the two ions is similar. At pH 2.0–4.5, metal ions interact primarily with carbonyl groups. With the increase of pH value, metal ions interacted with L-25 melanin.

**Table 1:** The effect of different metal ions on the stability of melanin.

Metal ions	Time (min)		
	0	60	120
Cu^2+^	1.903 ± 0.016*	1.856 ± 0.025*	1.814 ± 0.081*
Mg^2+^	1.969 ± 0.009**	1.944 ± 0.022**	1.873 ± 0.034*
Ca^2+^	1.848 ± 0.027*	1.842 ± 0.063	1.758 ± 0.019**
Zn^2+^	1.857 ± 0.026*	1.836 ± 0.075	1.801 ± 0.092
Mn^2+^	2.347 ± 0.013*	2.134 ± 0.045*	2.033 ± 0.067**
Al^3+^	2.196 ± 0.037*	2.042 ± 0.084	1.950 ± 0.063*
Fe^3+^	2.282 ± 0.039*	2.086 ± 0.095*	2.019 ± 0.061

*Differences are significant (*P* < 0.05).

**Differences are highly significant (*P* < 0.01).

### UV–visible absorption spectra

**Figure 2:**
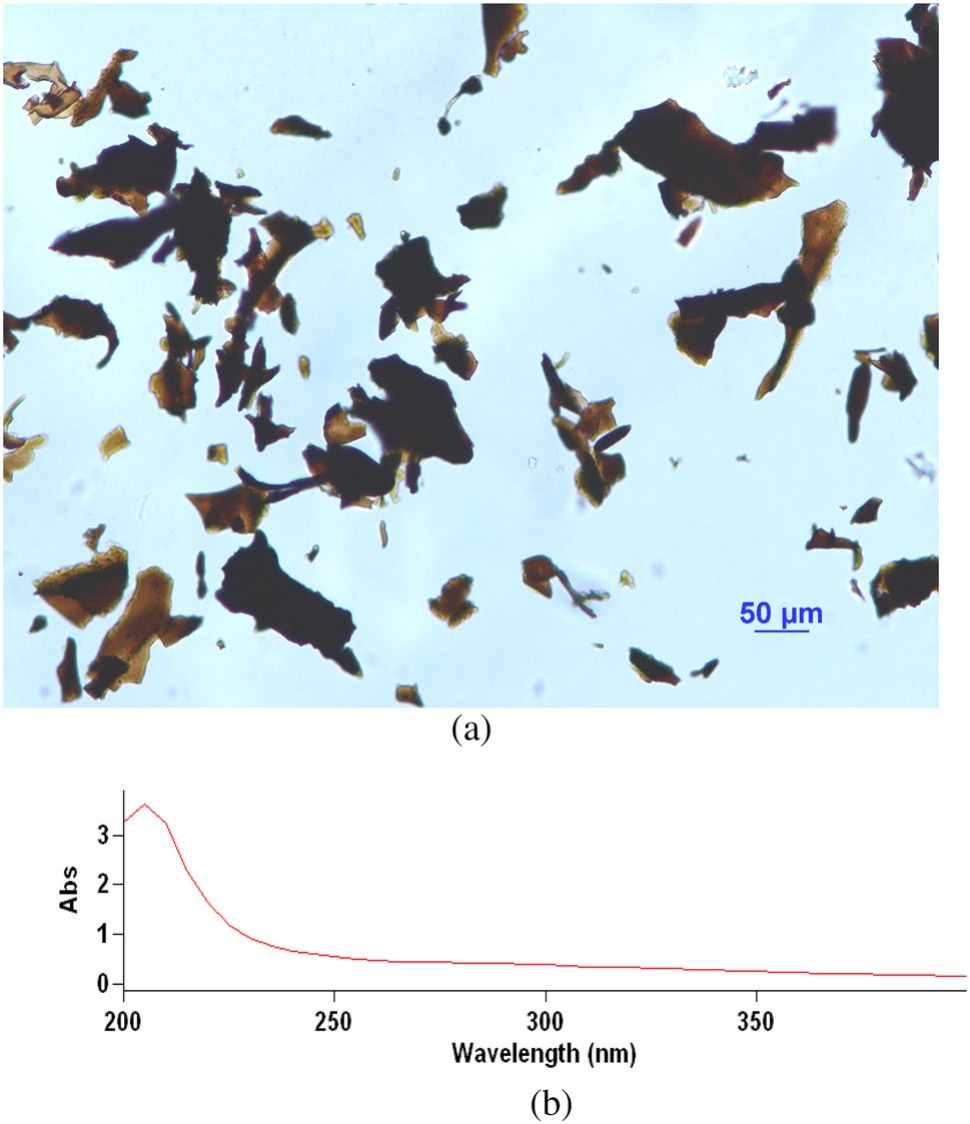
(**a**) The microscopic appearance of melanin from *S. reiliana.* (**b**) UV spectrum of L-25 melanin.

The maximum absorption of melanin was at 210 nm and decreased towards the visible region, which is a characteristic property of melanin (Fig. 2). This is because there are complex conjugated structures in the melanin molecules^19^. The decrease in absorption of L-25 melanin with the increase in wavelength is almost linear, and the log of absorbance against wavelength produces a linear curve with a negative slope of − 0.0031, which is one of the characteristics of melanin.

### IR spectra

**Figure 3:**
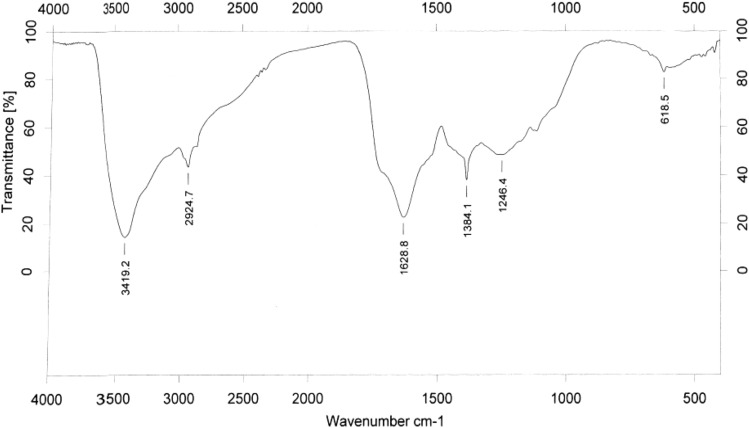
IR spectrum of melanins L-25 from *S. reiliana*.

Infrared absorption spectra revealed the most important spectral characteristics of L-25 melanin (Fig. 3). Based on previous studies, the infrared spectrum of L-25 melanin was analyzed in detail, and the results suggest the following peak assignments: the characteristic absorption of L-25 melanin at 3419 cm^−1^ was strong and broad corresponding to the O–H groups; There was an N–H stretch at 2924 cm^−1^ and a carboxyl group at 1629 cm^−1^. The strong infrared band in the range of 1380–1240 cm^−1^ indicated the presence of a pyran ring, and there was a C=O stretching vibration, in addition to alcoholic and phenolic hydroxyl groups in the range of 1100–1240 cm^−1^.

### Dynamic light scattering

L-25 melanin was prepared by RP-HPLC and the mass after concentration was about 20 mg. Melanin is an indole polymer and belongs to the group of eumelanin pigments. Studies have shown that eumelanin is derived from the black-brown insoluble melanin pigment subgroup^20^, which is derived from L-DOPA through the oxidative polymerization of 5,6-DL-hydroxy indole intermediates^13^.

**Figure 4:**
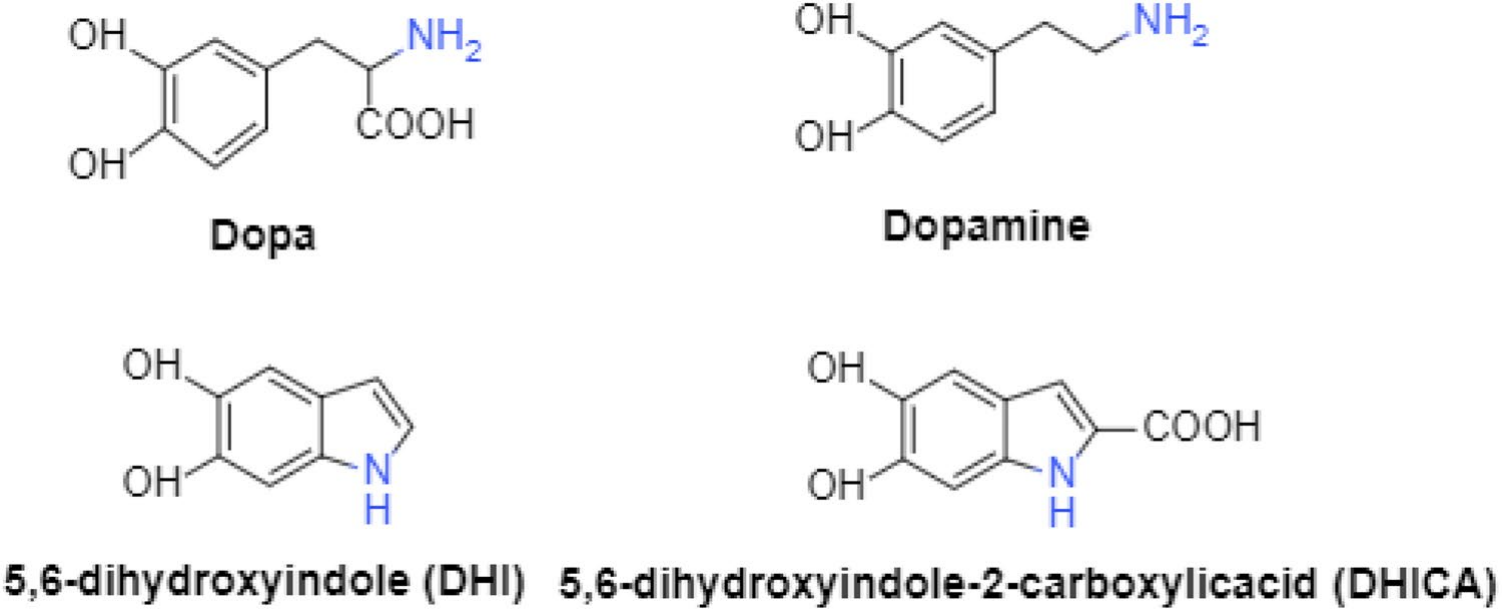
Melanin biosynthetic pathways.

When the maximum ultraviolet absorption wavelength was 215 nm, the dynamic light scattering spectrum showed that L-25 melanin had 7 main peaks (Fig. 4). After baseline separation by UPLC, the 7 component peaks were characterized by monitoring molecular ion characteristics. The mass spectrum results, the *m/z* ratio of the molecular ion peak and the ion fragments produced, and the QTOF-MS ion diagram are shown in supplementary materials (Table S9).

### Antioxidant activities

**Figure 5:**
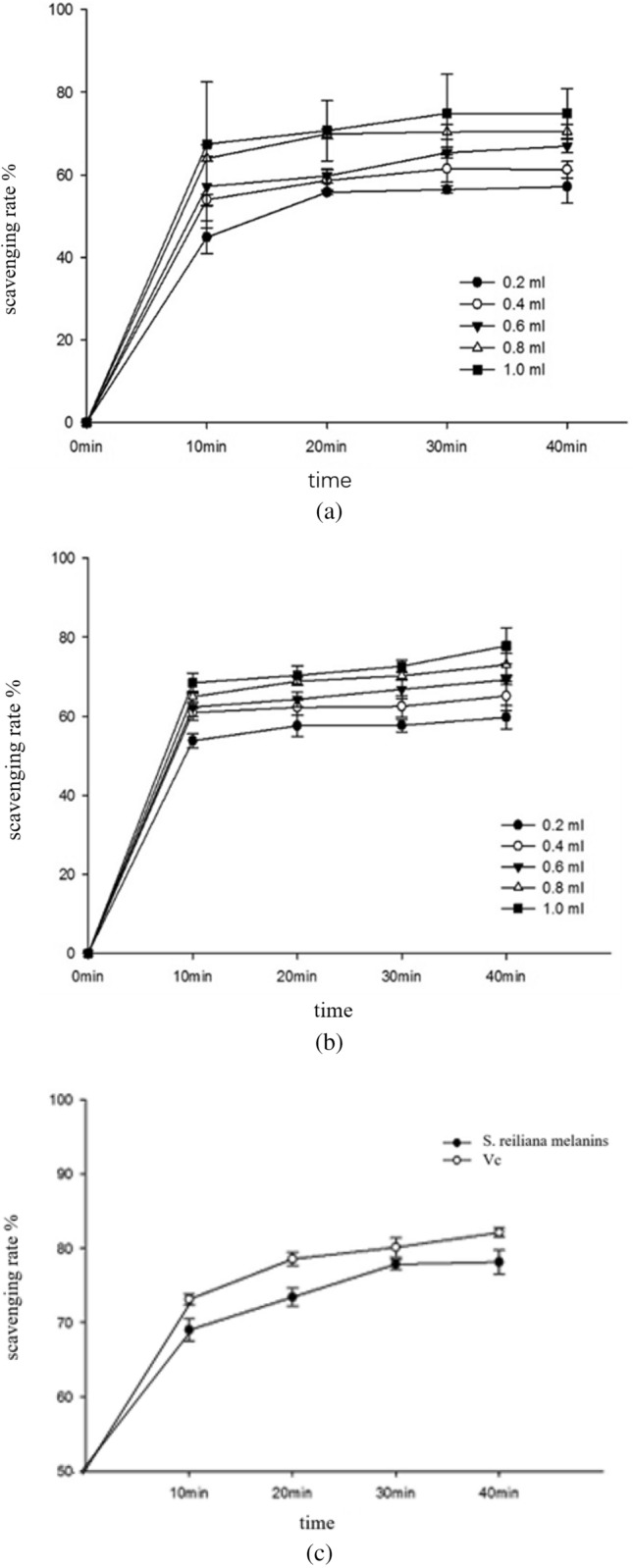
(**a**) DPPH^·^ scavenging ability of L-25 melanin; (**b**) DPPH^·^ scavenging ability of VC. VC, vinyl chloride. (**c**)Effect of reaction time on the ABTS^+·^ scavenging ability of VC and L-25 melanin.

The relatively stable organic radical DPPH has been widely used for the determination of the antioxidant activity of antioxidant compounds^21^. DPPH-containing solutions in ethanol appear purple in ethanol and have a strong absorbance at 517 nm. When antioxidants are paired with DPPH single electrons a gradual reduction of the color of the solution occurs^22^. The DPPH scavenging activities of L-25 melanin increased with increasing concentrations (Fig. 5) and showed a dose–response relationship. When the concentration of melanin was 0.5 mg/mL, the DPPH scavenging rates of L-25 melanin reached 72.35%. The activity of DPPH free radical scavenging by L-25 melanin was remarkably higher than that of melanin from black sesame (47.7%)^23^. Hydroxyl radicals readily react with biological molecules, such as amino acids, DNA, and proteins, which can lead to physiological disorders^24^. Therefore, it is necessary to find safe and effective antioxidants that scavenge hydroxyl radicals. The hydroxyl radical scavenging activities of L-25 melanin increased with increasing concentrations and exhibited a dose–response relationship. At a concentration of 0.4 mg/mL, the hydroxyl radical scavenging rate of L-25 melanin reached 76.86%. However, the ability of VC to scavenge DPPH free radicals was much greater than that of L-25 melanin. At the same time, a comprehensive comparison indicated that the DPPH free radical scavenging rate of VC was faster than that of L-25 melanin. In summary, the melanin from sorghum smut had the ability to remove DPPH free radicals but displayed only 20% of the removal activity of VC.

The ABTS radical cation is another commonly used organic radical to determine the antioxidant activity of single compounds or complex mixtures^25^. As shown in Fig. 5c, the effect of melanin and Vc on the ABTS free radical scavenging capacity was not significant with the extension of reaction time, the reaction rate of these two samples with ABTS free radical was fast and quickly reached equilibrium. The maximum clearance rate of melanin was 76.13%. This indicated that it was effective in scavenging ABTS free radicals.

### L25 melanin protects HepG2 cells against H_2_O_2_-induced oxidative damage

Cell proliferation is an important feature of life. It refers to the process of cell division through reactions such as DNA replication, RNA transcription, and protein synthesis. The growth of cancer cells can be evaluated by calculating the number of dividing cancer cells or the changes in the cell population, and this allows the determination of the effect of growth stimulation or inhibition of a sample^26^. This technology is widely used in many research fields such as tumor biology, molecular biology, pharmacokinetics, and pharmacology. It is not only critical for the study of the basic biological characteristics of cells, but also a basic method for analyzing cell states and studying genetic characteristics. The method can provide valuable information for exploring the pathogenesis, diagnosis, and treatment of a disease. Herein, the HepG2 cell line was used to evaluate the cytotoxic effect of L-25 melanin. As shown in Table 2, the survival rate of each mass concentration group is above 90%, and it had a certain proliferation effect at 200 mg/L. The results showed that melanin from sorghum smut had no obvious toxic effect on HepG2 cells in the concentration range of 10–400 mg/L (*P* < 0.05). Next, the effect of melanin at different concentrations and different times on the survival rate of HepG2 cells that were exposed to H_2_O_2_ was determined (Table 3). Compared with the control group, the cell survival rate of the H_2_O_2_ injury group had significantly decreased (*P* < 0.05). Compared with the H_2_O_2_ injury group, the cell survival rate of the sorghum smut melanin intervention group at a concentration of 10–100 mg/L was lower than that of the H_2_O_2_ injury group. However, when the sample concentration was 200 mg/L, the 6 h, 12 h, and 24 h treatments all showed significant protection (*P* < 0.05). The protective effect was greatest when the concentration of melanin reached 400 mg/L, and the survival rate reached 85%, which was 15% higher than the survival rate in the H_2_O_2_ injury model group. These results show that at concentrations between 200 and 400 mg/L sorghum smut melanin was able to protect HepG2 cells from oxidative injury induced by H_2_O_2_.

**Table 2:** Cytotoxicity of melanin on HepG2 cells.

Samples	Mass concentration (mg/L)	12 h A(x ± s)	Survival rate (%)
Normal cells	–	1.844 ± 0.014	100
Sorghum melanogaster melanogaster L-25–2	400	1.771 ± 0.061*	96
	300	1.678 ± 0.023*	91
	200	1.916 ± 0.009*	104
	100	1.714 ± 0.026*	93
	50	1.734 ± 0.048	94
	25	1.661 ± 0.037	90
	10	1.752 ± 0.019*	95

*P < 0.05.

**Table 3:** Protective effects of melanin on H_2_O_2_ induced HepG2 cells with different concentrations and time.

Samples	Mass concentration (mg/L)	12 h A(x ± s)			Survival rate (%)		
		6 h	12 h	24 h	6 h	12 h	24 h
Normal cells	–	0.733 ± 0.026	1.851 ± 0.052	1.938 ± 0.015	100	100	100
H_2_O_2_ damage	–	0.513 ± 0.014	1.258 ± 0.017	1.337 ± 0.009	70	68^a^	69^a^
Sorghum black powder fungus melanin L-25–2	400	0.624 ± 0.039	1.555 ± 0.028	1.581 ± 0.063	85^c^	84^b^	82^b^
	300	0.520 ± 0.056	1.296 ± 0.035	1.415 ± 0.028	71	70	73
	200	0.550 ± 0.009	1.388 ± 0.023	1.395 ± 0.009	73^b^	75^b^	72^b^
	100	0.439 ± 0.007	1.229 ± 0.065	1.221 ± 0.007	60	61	63
	50	0.432 ± 0.018	1.092 ± 0.017	1.24 ± 0.011	59	59	58
	25	0.425 ± 0.089	1.110 ± 0.023	1.221 ± 0.007	58	60	63
	10	0.410 ± 0.018	1.073 ± 0.017	1.104 ± 0.013	56	58	57

Compared with normal group, ^a^*P* < 0.01; compared with H_2_O_2_ damage model group, ^b^*P* < 0.05, ^c^*P* < 0.01.

As shown in Table 4, the values of ROS, MDA, and LDH in the H_2_O_2_ model group were 364.529 ± 4.106, 5.071 ± 0.262, and 5691.393 ± 22.896, respectively, and these values were significantly higher than those of the control group (*P* < 0.05). Compared with the model group, the three intervention groups at high, medium, and low concentrations of sorghum smut melanin displayed significantly reduced levels of ROS, MDA, and LDH in HepG2 cells that are damaged by hydrogen peroxide, and this effect was concentration dependent. It is inferred from these results that sorghum smut melanin can protect damaged HepG2 cells by reducing the levels of ROS, MDA, and LDH. Compared with the control group, the activity of SOD and GSH-Px in the H_2_O_2_ injury model group decreased significantly (*P* < 0.05) (Table 5). Compared with the H_2_O_2_ injury model group, the three intervention groups, treated with (high, medium, and low concentrations of sorghum smut melanin, respectively, displayed enhanced activities of the endogenous antioxidant enzymes SOD and GSH-Px, which confirms that sorghum smut melanin can protect HepG2 cells injury induced by H_2_O_2_. These results confirm the protective effect of melanin against cellular oxidative damage and its antioxidant activity.

**Table 4:** Effects of melanin on contents of ROS、MDA and LDH in H_2_O_2_ induced HepG2 cells.

Samples	Concentration (mg/L)	ROS (%)	MDA (μmol/L)	LDH (U/L)
Normal cells	–	101.983 ± 2.437	3.847 ± 0.159	935.715 ± 8.493
H_2_O_2_ model group	–	364.529 ± 4.106^a^	5.071 ± 0.262^a^	5691.393 ± 22.896^a^
Sorghum black powder fungus melanin L-25–2	200	318.734 ± 4.642	4.832 ± 0.268	5691.393 ± 22.896
	300	301.247 ± 2.968^b^	4.965 ± 0.304	5691.393 ± 22.896
	400	242.325 ± 3.712^b^	4.252 ± 0.273	5691.393 ± 22.896

Compared with normal group, ^a^*P* < 0.01; compared with H_2_O_2_ damage model group, ^b^*P* < 0.05, ^c^*P* < 0.01.

**Table 5:** Effects of melanin on contents of SOD and GSH-Px in H_2_O_2_ induced HepG2 cells.

Samples	Concentration (mg/L)	SOD (U/mL)	GSH-Px (U/mg)
Normal cells	–	43.153 ± 2.052	132.179 ± 3. 973
H_2_O_2_ model group	–	28.426 ± 1.637^a^	44.502 ± 6. 465^a^
Sorghum black powder fungus melanin L-25-2	200	32.514 ± 1.268	61.438 ± 5.236
	300	36.361 ± 2.034^b^	72.351 ± 4.137^b^
	400	37.618 ± 1.779^b^	90.169 ± 6.501^b^

Compared with normal group, ^a^*P* < 0.01; compared with H_2_O_2_ damage model group, ^b^*P* < 0.05, ^c^*P* < 0.01.

## Materials and methods

### Materials

*S. reiliana* was collected at the National Sorghum Improvement Center Base of the Liaoning Academy of Agricultural Sciences. The smut was harvested in mid-July. The bracts were removed, dried in the shade in a well-ventilated room, crushed, and the powder was stored until use. The extraction conditions of melanin has been reported by us previously (Lu and others, 2020): Ultrasonic power 245 W, temperature 50 °C, time 2 h, and pH 13 of NaOH extract.

The chemicals and solvents used for extraction and analysis in this study were all purchased from Sigma-Aldrich (St. Louis, MO, USA) or Solarbio (Beijing Solarbio Science and Technology Co., Ltd, Beijing, China).

### Solubility analysis

One milligram of L-25 melanin was added to test tubes containing 2 mL of distilled water, HCl, dilute sulfuric acid, NaOH, ammonia, ethanol, methanol, ethyl acetate, dichloromethane, DMSO, acetone, or petroleum ether. The sample was mixed with each solvent and incubated for 3 h, after which the solubility of L-25 melanin was measured.

### The effect of pH value on the stability of melanin

L-25 melanin (0.5 mg) was dissolved in ten milliliters of 0.1 mol/L NaOH or HCl solution with pH values of 3.0, 5.0, 7.0, 9.16, and 10.83 were prepared. The color change of the solution was observed, and the absorbance value (A) was measured at 215 nm.

### Light stability

A total of 1 mg of L-25 melanin was dissolved in 20 mL of 0.1 mol/L NaOH solution, then divided it into 2 parts. One part was placed under natural light for 4 days and samples were taken every day. The second part was placed under UV light for 30 min or 60 min, and the absorbance value A was measured at 215 nm.

### Effect of microwaving on the stability of melanin

L-25 melanin (50 mL) was prepared and heated in a microwave for different times (0–30 min). After cooling, the absorbance value A was measured at 215 nm.

### Oxidation–reduction properties

Various amounts of L-25 melanin solution were mixed with various volumes of vinyl chloride (VC), H_2_O_2_, Na_2_SO_3_, and sodium benzoate and added to 100 mL of water. After mixing, the solutions were placed in the dark for 1 h. Subsequently, the light absorption value A was measured at 215 nm.

### Metal ion stability

A 0.5 mg sample of L-25 melanin was dissolved in 10 mL of a 0.1 mol/L NaOH solution containing 0.01 mol/L of metal ions (Ca^2+^, Fe^2+^, Zn^2+^, Na^+^, Al^3+^, K^+^, Mg^2+^, Fe^3+^, and Cu^2+^) to explore the effect of metal ions on the stability of L-25 melanin. Samples were taken every 12 h, and their absorbance was measured at 212 nm.

### Detection of melanin complex with Cu^2+^ and Fe^3+^ by infrared spectroscopy

A sample of L-25 melanin (25 mg in 100 mL) was dissolved in 1 mol/L NaOH and filtered through a 0.45 μm microporous filter membrane. The solution was evenly divided into two parts. One part was mixed with 25 mL of a 0.02 mol/L CuSO_4_ solution. The other part was mixed with 25 mL of a 0.02 mol/L FeCl_3_ solution. Both mixtures were left for 12 h. Next, the pH was adjusted to 2.0, 3.0, 4.0, or 4.5 using 1 mol/L HCl, and the mixtures were left standing for 12 h. The acidified solution was stewed, centrifuged, and the precipitate was dried under vacuum.

### UV–Vis spectrum

The L-25 melanin was dissolved in 0.1 mol/L NaOH at a final concentration of 0.05 mg/mL. The UV–visible absorption spectrum of the L-25 melanin was scanned in the wavelength range of 200–800 nm using a UV–visible spectrophotometer (Unico Instrument Co. Ltd.) with 0.1 mol/L NaOH as the reference.

### Fourier-transform infrared spectroscopy

The melanin samples were mixed with KBr (1:100 w/w) and homogenized. The mixture of melanin and KBr was pressed into a tablet and analyzed by Fourier-transform infrared spectroscopy (Bruker VERTEX 80) in the scanning range of 4000–400 cm^−1^.

### Reversed-phase-HPLC preparation and separation conditions

The melanin sample was analyzed by reversed-phase using a Chromatograph CX-3000. The detector was a CX-3000. The analytical column was a C18 (4.0 × 250 mm). Mobile phase A was methanol and mobile phase B was water. The stepwise elution was as follows: 0 min 100% B, 20 min 30% A, 70% B, 50 min 100% A. The column temperature was 30 °C. The flow rate was 14 mL/min. The injection volume was 100 μL.

### Dynamic light scattering

Dynamic light scattering (UPLC-QTOF-MS) analysis was performed using Shimadzu’s liquid chromatography system and a triple TOF 5600 mass spectrometer (AB Sciex) equipped with an electrospray interface. Liquid chromatography conditions were as follows: the chromatographic column was an SB-Aq C18 column (2.7 μm), dimensions 3.0 × 100 mm (Agilent). The column was operated at 40 °C. Pump A contained 0.05 mol/L ammonium formate, pump B contained acetonitrile. The elution flow rate was 1.0 mL/min. The elution was as follows: 0–8 min, 10–40% B; 8–14 min, 40–60% B; 14–18 min, 60–70% B; 19–26 min, 70–95% B. The data was collected at 520 nm. Melanin (1 mg) was dissolved in 1 mL of methanol. Mass spectrometry analysis conditions were as follows: the injection volume of 1 μL was passed in the negative ion mode. The ionization source conditions were: atomization temperature 600 °C, atomization voltage 4.5 kV, DP-80, full scan spectrum in the range of 80–1500 *m/z*, cycle time was 0.2 s.

### DPPH free radical scavenging ability measurement

Antioxidant activities of substances are mainly assessed by the determination of free radical scavenging ability using cell models and chemical methods, of which the 2,2-diphenyl-1-picrylhydrazyl (DPPH^·^) method is the most commonly used. The experimental method was as reported in Zheng et al. (2015). Briefly, two test solutions were prepared for sorghum black powder, melanin, and VC, with mass concentrations of 2 mg/mL and 0.4 mg/mL, respectively. DPPH powder (5.92 mg) was dissolved in absolute ethanol in a 250 mL brown volumetric flask. The solution (6 × 10^–5^ mol/L) was stored in a dark place until use. The DPPH free radical solution (2 mL) was added to different amounts of sorghum smut melanin sample solution (0.2, 0.4, 0.6, 0.8, and 1.0 mL) and each sample was brought to 4 mL with absolute ethanol. The samples were shaken well, and diluted with deionized water after 10 min. The absorbance of each solution was measured at 517 nm at different times. As a reference, the sample solution was replaced with an equal volume of deionized water, so that the detected system only contained DPPH radicals and absolute ethanol. In an additional control, the DPPH radical solution was replaced with an equal volume of water. Distilled water was used as a control, V_c_ was used as positive control. The absorbance was measured using an ultraviolet spectrophotometer.

The calculation formula of the DPPH free radical scavenging rate was as follows:

$$K = \left( {1 - \frac{{A_{i} - A_{j} }}{{A_{c} }}} \right) \times 100\%$$

where K represents the scavenging rate of free radicals; *A*_*i*_ represents the absorbance of the DPPH free radical solution after adding the sample to be tested; *A*_*j*_ represents the absorbance of the sample without DPPH radical solution; *A*_*c*_ represents the absorbance without adding the sample to be tested, i.e., only the absorbance of the DPPH radical solution.

The experiment was conducted as previously described^27^ and the preparation of the different experimental solutions was as follows: An 2,2′-azino-bis(3-ethylbenzothiazoline-6-sulfonic acid (ABTS) + ·stock solution (7 mmol/L) was prepared by dissolving the ABTS free radicals in 2.45 mmol/L potassium persulfate and stored away from light at room temperature for 12–16 h. This stock solution was stable for 3–4 days.

The ABTS+ ·determination solution was prepared by diluting the ABTS^+·^ stock solution with absolute ethanol until its absorbance A reached 0.700 ± 0.020 at the maximum wavelength of 734 nm. Specifically, 40 μL of sorghum smut melanin sample test solution was added to 4 mL ABTS free radical test solution. The mixture was shaken for 30 s and the absorbance A was measured at the maximum absorption wavelength of 734 nm after various time intervals. Distilled water was used as a control, *V*_*c*_ was used as positive control.

### In vitro assays of cytotoxicity and proliferation

The effect of melanin on cytotoxicity and proliferation was measured using the thiazole blue (3-(4,5-dimethyl-2-thiazolyl)-2,5-diphenyl tetrazolium bromide (MTT) method, which is widely used for cell viability studies (Cao et al. 2017). Specifically, growing HepG2 cells were selected for our viability studies. The cells (100 μL) were suspended in growth media and inoculated into 96-well plates (9000 cells per well). Following the addition of PBS (150 μL) to each well the cells were incubated in a CO_2_ incubator for 24 h to make the cells adhere to the wall. Subsequently, the prepared melanin samples from the sorghum smut fungus were added to each well of the 96-well plate according to the set concentration, placed into the incubator, and cultured for another 24 h. Then, the MTT solution (0.5 mg/mL) was added to each well followed by a 4 h incubation. Then, the medium was aspirated and DMSO (150μL/well) was added, the plate was placed on a shaker and shaken well for 15 min, until complete color development. Finally, the 96-well plate was placed into a microplate reader and the absorbance at 490 nm was read. In the experiment, the growth rate of HepG2 cells in the control group without melanin was recorded as 100% and the survival rate of HepG2 cells in the remaining groups was calculated according to the following formula:

Cell survival rate (%) = (OD experimental group-OD blank group)/(OD control group-OD blank group) × 100%.

The melanin-treated cells were washed with PBS and trypsinized using 0.25% Trypsin and 0.02% EDTA. The cell suspension was centrifuged for 5 min at 4000 r/min, the cells were washed using PBS, and lysed with cell lysis buffer (150 mmol/L Tris–HCl, pH 8.0, 150 mmol/L NaCl, 1 mmol/L EDTA, 1% TritonX-100), followed by centrifugation at 4 °C for 1 h. The above clear liquids were used as samples and intracellular ROS, MDA, and LDH content was determined using a kit according to the manufacturer’s instructions. Intracellular SOD and GSH-Px activities were determined using a kit according to the manufacturer’s instructions. The SOD activity unit was defined as one nitrite unit when the SOD inhibition rate reached 50% per milligram of tissue protein in a 1 mL reaction mix; the GSH-Px activity unit was defined as the reduction of non-enzymatic reactions per milligram of protein per minute, to reduce the GSH concentration in the reaction system by 1 μmol/L. Protein quantification was determined by Coomassie Brilliant Blue colorimetry.

### Statistical analysis

All experiments were repeated at least three times, and the results were expressed as mean ± standard deviation. The statistical significance was analyzed using SPSS 22.0 software, and *P* < 0.05 was considered statistically significant. Origin 7.5 software was used to produce graphs.

## Conclusion

In this work, we systematically studied the physicochemical properties and the in vitro antioxidant and antiproliferative activity of L-25 melanin extracted from *S. reiliana*. The results showed that the L-25 melanin can be defined as DOPA-melanin with a maximum UV absorption at 215 nm. L-25 melanin has a good thermal stability and light resistance, good solubility under alkaline conditions, and L-25 melanin was stable in the presence of most metal ions. It also had significant antioxidant activity. The results on antioxidant and anti-proliferative activities have shown that melanin of Sorghum has moderate antioxidant activity in melanoma cells. Our cytotoxicity experiments showed that the melanin of *S. reiliana* protected HepG2 cells against H_2_O_2_-induced damage. The melanin can protect the damaged HepG2 cells by reducing the ROS, MDA, and LDH contents. In addition, compared with the H_2_O_2_ injury model group, three intervention groups, with high, medium, or low concentrations of melanin could enhance the activities of endogenous antioxidant enzymes SOD and GSH-Px, which confirmed the protective effect and antioxidant activity of melanin from S. reiliana against H_2_O_2_-induced oxidative damage in HepG2 cells. In the future, additional studies will be carried out on L-25 melanin to clarify its distribution and metabolism in vivo, and its applications in food and biomedical fields.

## Supplementary Information

Supplementary Information.
