# Supplementary material for: Characterization of the physicochemical properties, antioxidant activity, and antiproliferative activity of natural melanin from S. reiliana
Source: Sci Rep. 2022 Feb 8;12:2110. doi: 10.1038/s41598-022-05676-z (PMC8825793; doi:10.1038/s41598-022-05676-z)
Supplement: Supplementary file 1 — Supplementary Information. [file 41598_2022_5676_MOESM1_ESM.docx]

Table S1 The solubility of melanin

| Solvent | Dissolved volume /g | Color | Solubility |
| --- | --- | --- | --- |
| Distilled water | 0.1204 | Colorless transparent | insoluble |
| HCl | 0.0012 | Colorless transparent | insoluble |
| Dilute sulfuric acid | 0.0015 | Colorless transparent | insoluble |
| NaOH | 21.3486 | Dark brown | Soluble |
| Ammonia | 16.7217 | Dark brown | insoluble |
| Ethanol | 0.0047 | Colorless transparent | insoluble |
| Methanol | 0.2521 | Light Brown | slightly soluble |
| Ethyl acetate | 0.0037 | Colorless transparent | insoluble |
| Dichloromethane | 0.0318 | Colorless transparent | insoluble |
| DMSO | 0.3156 | Light Brown | slightly soluble |
| Acetone | 0.0612 | Colorless transparent | insoluble |
| Petroleum ether | 0.0014 | Colorless transparent | insoluble |

Table S2 Effects of PH value on the stability of the melanin

| pH | Absorbance value | Color | Sedimentation |
| --- | --- | --- | --- |
| 3 | 0.078±0.035 | Light brown | ** |
| 5 | 0.098±0.049 | Light brown | * |
| 7 | 0.325±0.024 | brownish red | - |
| 9.16 | 0.913±0.074 | dark brown | - |
| 10.83 | 0.908±0.067 | dark brown | - |

Note: "*" indicates the degree of precipitation of the pigment solution; "-" indicates that no precipitation is produced in the pigment solution.

Table S3 The effect of light on the stability of melanin

| Natural Light （d） | | | | | |  | | Ultraviolet light （min） | |
| --- | --- | --- | --- | --- | --- | --- | --- | --- | --- |
|  | 0 | 1 | 2 | 3 | 4 | 0 | 30 | | 60 |
| A | 0.851± | 0.858± | 0.873± | 0.882± | 0.885± | 0.849± | 0.863± | | 0.870± |
|  | 0.006***** | 0.013***** | 0.009****** | 0.027 | 0.015 | 0.031 | 0.009***** | | 0.028****** |

Note: * Differences are significant (P < 0.05); ** Differences are highly significant (P < 0.01).

Table S4 The effect of microwave on the stability of melanin

| Microwave（min） | 0 | 5 | 10 | 15 | 20 | 25 | 30 |
| --- | --- | --- | --- | --- | --- | --- | --- |
| A | 0.957±0.013***** | 0.974±0.017 | 0.956±0.024 | 0.942±0.027 | 0.947±0.021 | 0.951±0.018***** | 0.953±0.025***** |

Note: * Differences were significant (P < 0.05).

Table S5 The effect of H_2_O_2_ on the stability of melanin

| H_2_O_2_（mL） | 0 | 2 | 4 | 6 | 8 | 10 |
| --- | --- | --- | --- | --- | --- | --- |
| 1 h | 0.872±0.006****** | 0.897±0.023***** | 0.876±0.056 | 0.885±0.019****** | 0.878±0.008***** | 0.892±0.027***** |
| 2 h | 0.872±0.006****** | 0.836±0.043 | 0.841±0.032***** | 0.843±0.041***** | 0.836±0.027***** | 0.825±0.006****** |

Note: * Differences are significant (P < 0.05); ** Differences are highly significant (P < 0.01).

Table S6 The effect of Na2SO3 on the stability of melanin

| Na_2_SO_3_（mL） | 0 | 1 | 2 | 3 | 4 |
| --- | --- | --- | --- | --- | --- |
| 1 h | 0.835±0.024***** | 0.712±0.015***** | 0.503±0.101***** | 0.472±0.027****** | 0.458±0.004****** |
| 2 h | 0.835±0.024 | 0.432±0.038***** | 0.311±0.047***** | 0.152±0.039***** | 0.081±0.028****** |

Note: * Differences are significant (P < 0.05); ** Differences are highly significant (P < 0.01).

Table S7 The effect of Vc on the stability of melanin

| Vc（mL） | 0 | 1 | 2 | 3 | 4 |
| --- | --- | --- | --- | --- | --- |
| 1h | 0.887±0.013***** | 0.845±0.034***** | 0.836±0.043 | 0.845±0.023***** | 0.835±0.013****** |
| 2h | 0.885±0.035***** | 0.856±0.042 | 0.872±0.027***** | 0.816±0.037 | 0.826±0.015****** |
| 4h | 0.882±0.015****** | 0.828±0.034***** | 0.838±0.035****** | 0.824±0.019****** | 0.834±0.027***** |

Note: * Differences are significant (P < 0.05); ** Differences are highly significant (P < 0.01).

Table S8 The effect of sodium benzoate on the stability of melanin

| Sodium benzoate（mol/L） | 0 | 0.001 | 0.002 | 0.003 | 0.004 |
| --- | --- | --- | --- | --- | --- |
| 1h | 0.889±0.013****** | 0.793±0.034***** | 0.816±0.023 | 0.806±0.023***** | 0.828±0.041***** |
| 2h | 0.887±0.016****** | 0.824±0.016****** | 0.858±0.017***** | 0.848±0.026***** | 0.814±0.024***** |
| 4h | 0.882±0.023***** | 0.856±0.018***** | 0.727±0.014****** | 0.818±0.031***** | 0.836±0.017***** |

Note: * Differences are significant (P < 0.05); ** Differences are highly significant (P < 0.01).

Table S9 Tentative identification of the melanin compositions from L-25-2

| Serial number | [M-H]^-^ (*m/z*) | Possible attribution | Quasi-ionic peak time(min) |
| --- | --- | --- | --- |
| 5 | 311.1961 | C_14_H_23_N_4_O_4_（Ke，2006） | 22.400 |
| 6 | 325.2134 | C_20_H_25_N_2_O_2_ （Ke，2006） | 22.842 |
| 7 | 339.2302 | C_17_H_27_N_2_O_5_ （Ke，2006） | 23.215 |
| 1 | 677.5656 | C_17_H_27_N_2_O_5_, C_8_H_9_NO_2_, C_9_H_5_NO_4_（Xu，2006） | 13.188 |
| 2 | 790.6635 | 677.5656+113.0973 | 14.151 |
| 3 | 903.7575 | 790.6635+113.0940 | 14.897 |
| 4 | 1016.8587 | 903.7575+113.1012 | 15.511 |


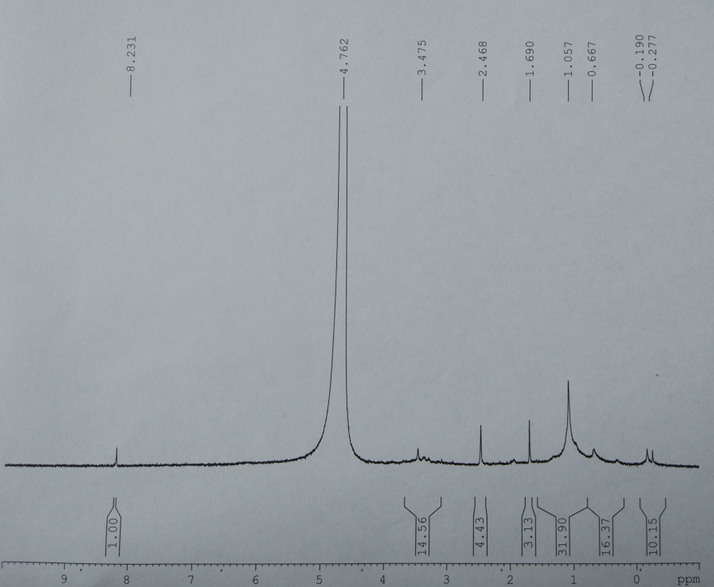


Fig.S1 ^1^H NMR spectrum of melanins L-25-2 from *S. reiliana*
